# The impact of dental status on perceived ability to eat certain foods and nutrient intakes in older adults: cross-sectional analysis of the UK National Diet and Nutrition Survey 2008–2014

**DOI:** 10.1186/s12966-019-0803-8

**Published:** 2019-05-14

**Authors:** Sinead Watson, Laura McGowan, Leigh-Ann McCrum, Christopher R. Cardwell, Bernadette McGuinness, Ciaran Moore, Jayne V. Woodside, Gerald McKenna

**Affiliations:** 1 Centre for Public Health, School of Medicine, Dentistry and Biomedical Sciences, Institute of Clinical Science Block A, Queen’s University Belfast, Belfast, BT12 6BJ UK; 2 Institute for Global Food Security, School of Biological Sciences, Queen’s University Belfast, Belfast, BT9 5BN UK

**Keywords:** Older adults, Nutrient intake, Nutritional status, Oral health, Dental status

## Abstract

**Background:**

Many factors determine dietary intake in older adults, including physical health, psychological well-being and socio-economic status. Dental status may also be important. The aim was to examine how dental status impacts perceived ability to eat to certain foods, nutrient intake and nutritional status in UK older adults.

**Methods:**

Data collected by the National Diet and Nutrition Survey Rolling Programme was analysed. A 4-day food diary assessed dietary intake, while a Computer Assisted Personal Interview collected socio-demographic, health behaviour and oral health information. Participants aged 65 years and over (*n* = 1053) were categorised into three groups according to their dental status: edentate with dentures (E-DEN, *n* = 292), dentate with dentures (D-DEN, *n* = 305) or dentate with no dentures (DEN, *n* = 456). A total of 515 participants provided a blood sample that was used to assess nutrient concentrations including vitamin B12, vitamin C, ferritin, vitamin B6 (pyridoxal-5-phosphate, PLP), retinol, β-carotene and 25-hydroxyvitamin D (25-OH-D). Multiple regression methods were performed to examine cross-sectional associations between dental status, food selection, nutrient intake and nutritional status.

**Results:**

Both E-DEN and D-DEN groups, compared with the DEN group, were more likely to report difficulty eating apples, raw carrots, lettuce, nuts, well-cooked steak and crusty bread (*P* < 0.01). No group differences were observed in perceived ability to eat sliced bread, sliced cooked meats and cheese. The E-DEN group compared with the DEN group had lower mean daily intakes of omega 3 fatty acids (*P* = 0.006), non-starch polysaccharides (*P* = 0.001), β-carotene (*P* = 0.001), folate (*P* = 0.001), vitamin C (*P* = 0.008), magnesium (*P* < 0.001) and potassium (P < 0.001), and had lower plasma vitamin B6 PLP (*P* = 0.001), vitamin C (*P* = 0.009) and β-carotene (*P* = 0.015) concentrations, after adjusting for socio-demographic and health behavioural factors. Compared with the DEN group, the D-DEN group did not have lower nutrient intakes or lower blood nutrient concentrations.

**Conclusions:**

Within this sample of older adults, impaired dental status appears to influence food selection, and intake of important nutrients. Future research should focus on developing dental interventions coupled with dietary counselling to encourage the adoption of healthy eating habits in this high-risk population group.

## Introduction

The proportion of older adults in the UK is increasing [1]. This poses several challenges to health and social care systems. For instance, consequences of an ageing population include increased incidence and prevalence rates of cardiovascular diseases, cancer, chronic respiratory diseases and dementia [2]. Diet plays an integral part in the prevention of age-related diseases. Older adults in the UK, however, are not meeting important dietary recommendations [3–5].

Dietary intake in older adults is influenced by an array of factors including, socio-economic status, living circumstances, physical health, and psychological wellbeing [6]. Dental status is also a potentially important factor [6]. Older adults are susceptible to tooth loss and a large number are edentate [7], which can impair masticatory ability. Consequently, reduced masticatory ability may impact dietary eating habits, such as avoiding tough foods that are high in fibre including fruit, vegetables and nuts [8–10]. Reductions in key nutrients for optimal health and healthy aging may subsequently occur. Removable dentures are typically prescribed for improving masticatory ability. Denture wearers, however, may find that their biting and chewing ability are still less efficient than those with natural teeth; hence, food selection can still be restricted [11, 12].

A previous nationally representative diet and nutrition survey of UK older adults by Sheiham and colleagues [13, 14], found that free-living edentate adults, compared with dentate adults, had lower intakes of a variety of important nutrients, as well as lower levels of serum ascorbic acid and retinol. The aforementioned survey, however, was undertaken approximately two decades ago; hence, it does not reflect contemporary society’s dietary intake. Furthermore, the most recent UK Adult Dental Health Survey 2009 shows that the proportion of older adults with total tooth loss has fallen over the past few decades, giving rise to a partially dentate older population [7]. No recent national surveys have been conducted in the UK that have explored if dental status remains a key determinant of dietary intake in older adults. The fact that UK older adults are currently not meeting important dietary guidelines also warrants further investigation of this relationship.

The aim of the study was to examine how dental status impacts perceived ability to eat to certain foods, nutrient intake and nutritional status in UK older adults. It was hypothesised that older adults categorised as having the least favourable dental status would have lower nutrient intakes, lower blood nutrient concentrations and are less likely to achieve dietary recommendations compared with older adults with the most favourable dental status.

## Method

### Study design and population

The National Data and Nutrition Survey Rolling Programme (NDNS RP) is a continuous cross-sectional survey of dietary habits and nutrient intakes of a representative sample of children (aged ≥1.5 to 18 yrs.) and adults (≥19 yrs.) living in private households in the UK. The study commenced in February 2008 and has a yearly recruitment target of approximately 1000 people.

The current analysis used data collected between February 2008 and August 2014 (years 1–6 combined) [15]. The survey design and sampling methods are described in detail elsewhere [3, 4]. Briefly, participants were sampled from the Postcode Address File; a database that includes all addresses in the UK. All addresses were clustered into Primary Sampling Units (PSU), which are small geographical areas based on postcode sectors. Addresses were randomly selected from each PSU and were sent a letter describing the survey’s purpose. The survey encompassed two stages: stage 1 was the interviewer visit, which involved three face-to-face visits at the participants home to gather socio-demographic, dietary intake and health behaviour information, and stage 2, if permission was granted at stage 1, was the nurse visit to conduct physical measurements and to collect biological specimens.

Only adults aged ≥65 years were included in the current analysis (*n* = 1076). Within this sub-population, those who reported implausible energy intake (< 2512 or > 15,070 kJ/d [< 600 or > 3600 kcal/d] for women and < 3349 or > 17,581 kJ/d [< 800 or > 4200 kcal/d] for men) (*n* = 8); those who reported having no natural teeth and not wearing a denture (*n* = 10), and those with missing covariates (*n* = 5) were excluded. The final analytical sample was 1053 participants. Of the 1053 participants, 515 provided an overnight fasting blood sample, which was analysed for biochemical markers of nutritional status.

### Dietary intake

Dietary intake was assessed using an estimated four-day food diary, which involved participants recording everything they consumed over four consecutive days at home and away from home, including weekends and weekdays. Food/drink portion sizes were estimated using household measures (e.g. two slices of thick bread) or using weights from food/drink packaging. Trained interviewers checked the food diaries for inaccuracies and omissions during the collection period and no later than three days after the final day of recording. Within this subpopulation 1029 (98%) participants completed four diary days, while 24 (2%) participants completed three diary days. The in-house dietary assessment software, Diet in Nutrients Out (DINO), provided estimates for energy and nutrient intakes from food/drink sources (excluding supplements) using the Department of Health’s NDNS Nutrient Databank [16]. The majority of nutrients selected for the current analysis (see Tables 3 and 4) have been identified by previous studies as nutrients of concern for individuals with impaired masticatory ability [8–10] and for older adults [3–5].

Older adults in the UK are currently not meeting important dietary recommendations [3–5], notably the five portions per day (80 g is one portion) of fruit and vegetables, the 18 g/d of non-starch polysaccharides (NSP) and the 140 g/w of oily fish. Furthermore, men aged 65 years and over are likely to exceed the 70 g/d of red and processed meat [3–5]. In the current analysis mean daily intakes of the above foods, as well as mean NSP intake were compared with the above recommended levels, and the relationship with dental status explored.

### Oral health

Oral Health information was collected by a Computer Assisted Personal Interview (CAPI). Participants were asked about their dental status, i.e. if they had any natural teeth (dentate) or not (edentate), and if they wore dentures. A clinical oral examination was not completed. For the current analysis participants were categorised into three groups according to their dental status: edentate with dentures (E-DEN, *n* = 292), dentate with dentures (D-DEN, *n* = 305) or dentate with no dentures (DEN, *n* = 456).

To assess the impact of dental status on eating ability, participants were asked to rate their perceived level of difficulty they had when eating (biting, chewing and swallowing) a list of 12 food items, including sliced bread, crusty bread, cheese, tomatoes, raw carrots, cooked green vegetables, lettuce, apples, oranges, nuts, sliced cooked meat and well-cooked steak. In survey years 1–5 participants were asked whether they could eat each of the listed foods with no difficulty, with some difficulty, or if they could not eat them at all. In year 6 participants were asked if they had any difficulty eating each of the listed food items. A binary outcome for each food listed above was computed for analysis (eat with some difficulty/could not eat all *vs*. no difficulty).

### Biochemical measures of nutritional status

Underreporting of energy intake has been identified in the NDNS RP in all age groups by a sub study using the Doubly Labelled Water (DLW) technique. Thus, to corroborate any recognised associations between dental status and the self-reported nutrient intakes, a number of nutritional blood biomarkers were selected for the current analysis. A total of 515 participants provided an overnight fasting blood sample. Reasons for not obtaining a blood sample included, medical reasons, i.e. the participant had a bleeding or clotting disorder or were taking anti-coagulant medication; for reporting being hepatitis B or HIV positive; for not consenting to have the blood sample taken; and due to the nurse not being able to find a suitable vein or the vein collapsing during the procedure.

The nutritional blood biomarkers selected for the current analysis included, serum vitamin B12 (*n* = 507), and plasma vitamin C (*n* = 476), ferritin (*n* = 510), vitamin B6 (pyridoxal-5-phosphate, PLP) (*n* = 496), retinol (*n* = 497), β-carotene (*n* = 500) and 25-hydroxyvitamin D (25-OH-D) (*n* = 499). Blood sample collection, processing, storage and analysis are described in detail in the NDNS RP published reports and appendices [3, 4]. Serum samples were assayed at Addenbrooke Hospital, Cambridge, and plasma samples were assayed at MRC, Human Nutrition Research.

### Anthropometric data

Height and weight were measured using a portable stadiometer to the nearest 0.1 cm, and weighing scales to the nearest 0.1 kg, respectively. Body mass Index (BMI) was calculated (weight divided by height squared) using height (m^2^) and weight (kg) measurements. Waist circumference (cm) was measured using an insertion tape measure.

### Covariates

Socio-demographic variables, captured by CAPI, included age, gender, socio-economic status (SES) and living status. Socio-economic status (SES) was determined based on the National Statistics Socio-Economic Classification System (NSSEC) [17] of the household reference person (HRP), (a person in whose name the property is owned or rented) with the highest income, not necessarily the person who completed the food diary etc. The NSSEC system is based on most recent/current occupational status. Three occupational classes were used for the current analysis: higher occupations, representing higher managerial, administration and professional occupations; medium occupations, representing positions in clerical, sales, and intermediate technical occupations; and lower occupations, representing routine and manual occupations. Those who have never worked were included within the lower occupation group.

Household size was used to determine living status (living alone or living with others). Health behaviour indicators included cigarette smoking status (current smoker, ex-regular smoker and never regularly smoked), and nutritional supplement use (taking supplements or not taking supplements).

### Statistical analysis

All analyses were preformed using SPSS for Windows version 25.0. To examine the differences in characteristics of the sample according to dental status, Analysis of Variance (ANOVA) tests (continuous variables) and χ^2^ tests (categorical variables) were carried out.

Logistic regression analysis, with ability to eat (eat with some difficulty/could not eat all vs. no difficulty) as the outcome and dental status as an explanatory variable (using the DEN group, with remaining natural teeth and no dentures, as the reference category), was conducted to examine the impact of dental status on perceived ability to eat 11 selected food items. Odds ratios (ORs) and 95% confidence intervals (CIs) were calculated. An unadjusted analysis was conducted as well as an adjusted analysis in which models contained classic confounders including age and gender, as well as survey year (continuous variable). Analyses of cooked green vegetables are not presented because only a small number of individuals reported having difficulty eating cooked green vegetables, resulting in unstable estimates.

Multiple linear regression analysis, with nutrient intake (excluding nutritional supplements) as the outcome and dental status as an explanatory variable, was conducted to investigate the associations between dental status and nutrient intakes. The difference in mean nutrient intake (and 95% CIs) compared with the DEN group, which acted as the reference category, was calculated. An unadjusted analysis was conducted as well as an adjusted analysis. Potential confounders, identified from the literature, included age, gender, SES (low, med and high), living status (living alone or living with others), survey year (continuous variable), energy intake (kJ/d) and smoking status (current smoker, ex-regular smoker and never regular smoker). Similar analyses were conducted for biochemical measures of nutritional status. Nutritional supplement use (yes/no) was also adjusted for when analysing the relationships between dental status and the biochemical measures of nutritional status. Some of the nutrients derived from the food diaries and measured in blood were positively skewed; hence were natural log transformed for analysis (see Tables 4 and 6). These data were presented as geometric means (interquartile ranges), and ratios of geometric means (95% CIs).

Logistic regression analysis was conducted to examine the impact of dental status on adherence to four UK dietary recommendations. The outcome was adherence to four UK dietary recommendations and the explanatory variable was dental status (with the DEN group as the reference category). Dietary recommendations were for intakes of fruit and vegetables (5 portions of a variety of fruit and vegetables daily); red and processed meat (not exceed 70 g daily); oily fish (140 g per week) and NSP (18 g daily) [18, 19]. Unadjusted and adjusted analyses were conducted in which models contained socio-demographic variables (age, gender, SES and living alone), survey year, energy intake (kJ/d) and smoking status.

## Results

Sample characteristics are presented in Table 1. The mean (SD) age of the sample was 74.1 (6.94) years. According to BMI classifications [20, 21] the majority (677/918; 74%) of the sample were overweight or obese (BMI ≥25 kg/m^2^), and 7% (64/918) were considered malnourished (BMI < 20 kg/m^2^ if < 70 years or < 22 kg/m^2^ if > 70 years). Twenty-eight percent of the sample reported having no remaining natural teeth, and 57% of the sample reported wearing a denture. E-DEN and D-DEN participants were older (*P* < 0.001), were more likely to be female (*P* = 0.021), live alone (*P* < 0.001), and were shorter (*P* < 0.001). E-DEN participants were more likely to be from a lower SES group (*P* < 0.001), and DEN participants were more likely to report never smoking cigarettes (*P* = 0.001).

**Table 1:** Characteristics of community-dwelling ≥65 year olds according to dental status

Characteristic	Total		DEN		D-DEN		E-DEN		
	*n* = 1053		*n* = 456		*n* = 305		*n* = 292		
Socio-demographics	n	%	n	%	n	%	n	%	P^b^
Female	621	59	247	54	191	63	183	63	0.021
Age (yrs.)									
65–74	613	58	313	69	175	57	125	43	
75+	440	42	143	31	130	43	167	57	< 0.001
SES^a^									
High	346	33	186	41	110	36	50	17	
Medium	234	23	107	24	71	23	65	22	
Low	464	44	163	36	124	41	177	61	< 0.001
Living alone	521	50	190	42	163	53	168	58	< 0.001
Anthropometry	Mean	SD	Mean	SD	Mean	SD	Mean	SD	P^c^
Height cm (*n* = 933)	163.33	9.17	164.64	9.02	162.78	8.75	161.71	9.57	< 0.001
Weight kg [*n* = 959)	75.25	15.87	75.61	15.39	74.63	14.17	75.33	18.23	0.725
BMI kg/m^2^ (*n* = 918)	28.23	5.10	27.93	4.71	28.14	4.75	28.81	6.00	0.099
Waist circumference cm (*n* = 756)	97.29	14.05	96.48	14.16	97.02	12.64	99.03	15.22	0.119
Health behaviours	n	%	n	%	n	%	n	%	P^b^
Smoking status									
Current cigarette smoker	116	11	39	9	34	11	43	15	
Ex-regular cigarette smoker	396	38	151	33	123	40	122	42	
Never regularly smoked cigarettes	541	51	266	58	148	49	127	44	0.001
Takes a nutritional supplement	363	35	174	38	102	33	87	30	0.057

SES, socio-economic status. ^a^SES was determined based on the National Statistics Socio-Economic Classification System (NSSEC) of the household reference person: High SES includes higher managerial, administrative and professional occupations; medium SES includes intermediate occupations; low SES includes routine and manual occupations. Categorical data are presented as frequencies (%), and continuous data are presented as mean (SD). Significant differences between DEN, D-DEN & E-DEN groups for categorical data were analysed using ^b^Chi^2^ test, and for continuous data ^c^ANOVA tests were used

Participants who provided a blood sample for measurement of biochemical markers of nutritional status (*n* = 515) were younger (mean [SD]: 73.0 [6.40] vs. 75.1 [7.21] years; *P* < 0.001); were more likely from a higher SES (*P* = 0.002); were taller (mean [SD]: 164.0 [8.73] vs. 162.6 [9.56] cm; *P* = 0.020); had a lower BMI (mean [SD]: 27.9 [4.89] vs. 28.6 [5.29] kg/m^2^; *P* = 0.042) and a higher daily energy intake (mean [SD]: 7179.0 [2028.2] vs. 6714.8 [1828.0] kJ) compared to those who didn’t provide a blood sample.

The impact of dental status on perceived ability to eat a variety of selected foods is shown in Table 2. After controlling for age, gender and survey year, the E-DEN group compared with the DEN group were more likely to report having difficulty eating eight foods out of the 11 foods listed, including crusty bread, tomatoes, raw carrots, lettuce, apples, oranges, nuts, and well-cooked steak (*P* values < 0.001). For instance, the odds of not being able to eat/being able to eat with some difficultly crusty bread in the E-DEN group was 3 times that of the DEN group (adjusted OR = 3.05 95% CI 1.96, 4.77). Similar comparisons were observed between the D-DEN and DEN groups except for tomatoes (*P* = 0.221) and oranges (*P* = 0.096).

**Table 2:** The impact of dental status on perceived ability to eat various food types

Food type	Dental status	Could eat with some difficulty/could not eat at all			Unadjusted			Adjusted^a^	
		n	%	OR	95% CI	P	OR	95% CI	P
Sliced bread	DEN	5	1.1	Ref	Ref	Ref	Ref	Ref	Ref
	D-DEN	3	1.0	0.90	0.21, 3.79	0.885	0.78	0.18, 3.32	0.740
	E-DEN	9	3.1	2.87	0.95, 8.65	0.061	2.22	0.70, 7.04	0.174
Crusty bread	DEN	37	8.1	Ref	Ref	Ref	Ref	Ref	Ref
	D-DEN	61	20.0	2.83	1.83, 4.39	< 0.001	2.57	1.65, 4.01	< 0.001
	E-DEN	72	24.8	3.74	2.44, 5.74	< 0.001	3.05	1.96, 4.77	< 0.001
Cheese	DEN	7	1.5	Ref	Ref	Ref	Ref	Ref	Ref
	D-DEN	10	3.3	2.17	0.82, 5.78	0.119	2.07	0.78, 5.55	0.146
	E-DEN	11	3.8	2.52	0.97, 6.58	0.059	2.24	0.83, 6.05	0.113
Tomatoes	DEN	8	1.8	Ref	Ref	Ref	Ref	Ref	Ref
	D-DEN	10	3.3	1.90	0.74, 4.87	0.182	1.81	0.70, 4.65	0.221
	E-DEN	24	8.2	5.03	2.23, 11.37	< 0.001	4.62	1.99, 10.71	< 0.001
Raw carrots	DEN	49	10.7	Ref	Ref	Ref	Ref	Ref	Ref
	D-DEN	89	29.3	3.44	2.34, 5.06	< 0.001	3.30	2.23, 4.87	< 0.001
	E-DEN	117	40.8	5.72	3.92, 8.35	< 0.001	5.04	3.41, 7.45	< 0.001
Lettuce	DEN	6	1.3	Ref	Ref	Ref	Ref	Ref	Ref
	D-DEN	18	5.9	4.70	1.85, 11.99	0.001	4.57	1.79, 11.70	0.002
	E-DEN	23	7.9	6.41	2.58, 15.95	< 0.001	5.99	2.35, 15.21	< 0.001
Apples	DEN	51	11.2	Ref	Ref	Ref	Ref	Ref	Ref
	D-DEN	92	30.2	3.43	2.35, 5.02	< 0.001	3.31	2.26, 4.87	< 0.001
	E-DEN	121	41.4	5.62	3.87, 8.16	< 0.001	5.25	3.57, 7.73	< 0.001
Oranges	DEN	9	2.0	Ref	Ref	Ref	Ref	Ref	Ref
	D-DEN	13	4.3	2.21	0.93, 5.24	0.071	2.09	0.88, 4.97	0.096
	E-DEN	26	8.9	4.87	2.25, 10.56	< 0.001	4.35	1.96, 9.66	< 0.001
Nuts	DEN	53	11.6	Ref	Ref	Ref	Ref	Ref	Ref
	D-DEN	82	26.9	2.79	1.90, 4.09	< 0.001	2.63	1.79, 3.87	< 0.001
	E-DEN	104	35.7	4.22	2.90, 6.13	< 0.001	3.65	2.48, 5.38	< 0.001
Sliced cooked meats	DEN	8	1.8	Ref	Ref	Ref	Ref	Ref	Ref
	D-DEN	13	4.3	2.50	1.02, 6.11	0.044	2.23	0.91, 5.48	0.081
	E-DEN	11	3.8	2.19	0.87, 5.52	0.096	1.79	0.69, 4.65	0.234
Well-cooked steaks	DEN	50	11.0	Ref	Ref	Ref	Ref	Ref	Ref
	D-DEN	88	29.0	3.32	2.26, 4.87	< 0.001	3.07	2.08, 4.54	< 0.001
	E-DEN	103	35.5	4.46	3.05, 6.52	< 0.001	3.81	2.57, 5.65	< 0.001

Data analysed using logistic regression and presented as odd ratios (95% CI). DEN group was fixed as the reference category in each model. ^a^Adjusted for age, gender and survey year

Table 3 shows mean disparities in energy and nutrient intakes according to dental status. Compared with the DEN group, the E-DEN group had lower mean daily intakes of all the nutrients listed in Table 3 (unadjusted models) except for saturated fat (*P* = 0.328). Following adjustment for socio-demographic variables, energy intake, smoking status and survey year, the E-DEN group compared with the DEN group, had significantly lower mean daily intakes of omega 3 fatty acids (*P* = 0.006) and NSP (*P* = 0.001). The D-DEN group had significantly lower mean daily intakes of omega 3 fatty acids (*P* = 0.012) compared with the DEN group (unadjusted model); however, following adjustment this relationship was no longer significant. The E-DEN group and D-DEN group had significantly higher mean daily carbohydrate intakes compared with the DEN group following adjustment (*P* < 0.05).

**Table 3:** The relationship between dental status and daily nutrient intake

				Unadjusted			Adjusted^a^		
Nutrient	Dental status	Mean	SD	Mean difference	95% CI	P	Mean difference	SD	P
Energy kJ/d [1 kcal = 4.186 kJ)	DEN	7178.68	2000.11	Ref	Ref	Ref	Ref	Ref	Ref
	D-DEN	6939.84	1891.55	− 238.84	− 518.59, 40.91	0.094	−87.55	− 359.96, 184.86	0.528
	E-DEN	6574.01	1846.91	− 604.67	− 888.13, − 321.21	< 0.001	38.83	− 212.96, 290.61	0.762
Protein g/d	DEN	70.85	20.56	Ref	Ref	Ref	Ref	Ref	Ref
	D-DEN	69.20	18.12	−1.65	−4.41, 1.11	0.242	0.71	−1.13, 2.55	0.447
	E-DEN	63.46	17.33	−7.39	−10.18, − 4.59	< 0.001	− 1.73	−3.71, 0.26	0.089
Fat g/d	DEN	64.84	23.14	Ref	Ref	Ref	Ref	Ref	Ref
	D-DEN	62.25	21.81	−2.59	−5.81, 0.62	0.114	−0.77	− 2.42, 0.88	0.359
	E-DEN	60.02	20.88	−4.82	−8.08, − 1.56	0.004	0.04	−1.75, 1.82	0.968
Sat Fat g/d	DEN	25.24	10.56	Ref	Ref	Ref	Ref	Ref	Ref
	D-DEN	24.42	9.67	−0.82	−2.31, 0.67	0.279	−0.35	−1.31, 0.61	0.476
	E-DEN	24.48	10.33	−0.75	−2.26, 0.76	0.328	0.64	−0.40, 1.68	0.229
Mono Fat g/d	DEN	22.52	8.58	Ref	Ref	Ref	Ref	Ref	Ref
	D-DEN	21.57	8.19	−0.95	−2.13, 0.22	0.112	−0.19	−0.88, 0.50	0.588
	E-DEN	20.43	7.18	−2.09	−3.28, − 0.90	0.001	− 0.13	− 0.88, 0.62	0.743
Omega 3 fatty acids g/d	DEN	2.04	1.05	Ref	Ref	Ref	Ref	Ref	Ref
	D-DEN	1.86	0.98	−0.18	−0.33, − 0.04	0.012	− 0.10	− 0.23, 0.02	0.110
	E-DEN	1.63	0.86	−0.41	−0.56, − 0.27	< 0.001	− 0.19	− 0.32, − 0.05	0.006
Omega 6 fatty acids g/d	DEN	8.72	3.76	Ref	Ref	Ref	Ref	Ref	Ref
	D-DEN	8.27	3.58	−0.45	− 0.97, 0.07	0.090	− 0.10	− 0.48, 0.29	0.632
	E-DEN	7.54	3.25	−1.18	− 1.71, −0.66	< 0.001	−0.32	− 0.74, 0.10	0.134
Carbohydrate g/d	DEN	204.71	63.81	Ref	Ref	Ref	Ref	Ref	Ref
	D-DEN	202.84	60.70	−1.87	−10.69, 6.96	0.679	5.22	0.76, 9.69	0.022
	E-DEN	194.59	55.93	−10.12	− 19.07, − 1.18	0.027	6.80	1.97, 11.63	0.006
Total sugars g/d	DEN	92.94	39.15	Ref	Ref	Ref	Ref	Ref	Ref
	D-DEN	92.94	39.15	−0.53	−5.96, 4.90	0.848	2.29	−1.74, 6.33	0.265
	E-DEN	82.00	33.92	−10.94	−16.45, −5.44	< 0.001	−2.35	−6.71, 2.02	0.291
NSP g/d	DEN	14.31	4.98	Ref	Ref	Ref	Ref	Ref	Ref
	D-DEN	13.67	4.73	−0.64	−1.32, − 1.80	0.068	− 0.13	− 0.71, 0.45	0.664
	E-DEN	11.82	4.25	−2.50	−3.19, − 1.80	< 0.001	− 1.11	− 1.74, − 0.48	0.001

Sat Fat, saturated fat; Mono fat, monounsaturated fat; and NSP, non-starch polysaccharides. Data analysed using multiple regression. Data presented as means (SD), and unadjusted and adjusted differences in means (95% CI). The DEN group was fixed as the reference category in each model. ^a^Adjusted for age, gender, SES (low, med & high), living status (living alone or not living alone), energy intake (kJ/d), survey year and smoking status (current smoker, past regular smoker & never smoked)

The E-DEN group compared with the DEN group had significantly lower mean daily intakes of all the micronutrients listed in Table 4 except for retinol (unadjusted model). After adjustment, the E-DEN group compared with the DEN group, had significantly lower mean daily intakes of β-carotene (*P* = 0.001), folate (*P* = 0.001), vitamin C (*P* = 0.008), magnesium (*P* < 0.001) and potassium (*P* < 0.001). The D-DEN group had significantly lower mean daily intake of vitamin B6 (*P* = 0.037) and folate (*P* = 0.029) compared with the DEN group (unadjusted model); however, following adjustment there was little evidence of these associations. The D-DEN group had a significantly higher mean calcium intake compared with the DEN group after adjustment (*P* = 0.035).

**Table 4:** The relationship between dental status and daily micronutrient intakes

				Unadjusted			Adjusted^b^		
Nutrient	Dental status	Mean	SD	Mean difference	95% CI	P	Mean difference	95% CI	P
Retinol μg/d^a^	DEN	339.30	218.15, 443.08	Ref	Ref	Ref	Ref	Ref	Ref
	D-DEN	354.27	215.00, 465.56	−1.01	− 1.15, 1.12	0.834	1.07	−1.05, 1.21	0.239
	E-DEN	334.76	216.15, 465.71	1.04	−1.08, 1.18	0.495	1.04	−1.09, 1.18	0.509
β-carotene μg/d^a^	DEN	2364.80	1403.04, 4231.63	Ref	Ref	Ref	Ref	Ref	Ref
	D-DEN	2099.42	1182.22, 3723.48	−1.13	− 1.29, 1.00	0.081	−1.09	− 1.24, 1.05	0.214
	E-DEN	1637.74	718.21, 3724.93	1.44	−1.65, − 1.26	< 0.001	− 1.28	−1.48, − 1.11	0.001
Vitamin B6 mg/d	DEN	2.04	0.78	Ref	Ref	Ref	Ref	Ref	Ref
	D-DEN	1.93	0.66	−0.12	− 0.22, − 0.01	0.037	−0.06	− 0.14, 0.03	0.223
	E-DEN	1.87	0.78	−0.17	− 0.28, − 0.06	0.002	−0.04	− 0.14, 0.06	0.421
Vitamin B12 μg/d^a^	DEN	5.16	3.65, 7.15	Ref	Ref	Ref	Ref	Ref	Ref
	D-DEN	5.21	3.76, 6.88	1.01	1.06, 1.08	0.853	1.03	−1.03, 1.10	0.513
	E-DEN	4.70	3.49, 5.99	−1.08	− 1.16, − 1.01	0.028	− 1.02	− 1.10, 1.05	0.307
Folate μg/d	DEN	261.85	100.67	Ref	Ref	Ref	Ref	Ref	Ref
	D-DEN	246.95	82.59	−14.90	−28.30, − 1.49	0.029	− 5.69	− 17.01, 5.64	0.325
	E-DEN	219.17	88.35	−42.67	−56.26, − 29.09	< 0.001	− 20.48	− 32.74, − 8.23	0.001
Vitamin C mg/d	DEN	84.10	49.21	Ref	Ref	Ref	Ref	Ref	Ref
	D-DEN	81.50	47.32	−2.60	−9.40, 4.21	0.454	0.82	−5.59, 7.23	0.801
	E-DEN	63.33	42.41	−20.77	− 27.66, − 13.87	< 0.001	− 9.34	−16.28, − 2.41	0.008
Vitamin D μg/d^a^	DEN	2.83	3.65, 7.15	Ref	Ref	Ref	Ref	Ref	Ref
	D-DEN	2.69	3.76, 6.88	−1.04	−1.12, 1.03	0.282	−1.01	−1.08, 1.06	0.790
	E-DEN	2.39	1.62, 3.65	−1.14	− 1.22, − 1.06	< 0.001	− 1.06	− 1.13, 1.02	0.120
Iron mg/d	DEN	10.36	3.25	Ref	Ref	Ref	Ref	Ref	Ref
	D-DEN	10.01	3.25	−0.36	−0.82, 0.11	0.136	0.02	−0.32, 0.37	0.893
	E-DEN	9.03	3.13	−1.34	−1.81, − 0.86	< 0.001	− 0.35	− 0.73, 0.02	0.065
Calcium mg/d	DEN	826.60	291.27	Ref	Ref	Ref	Ref	Ref	Ref
	D-DEN	841.95	285.93	15.35	−26.73, 57.43	0.474	34.97	2.40, 67.53	0.035
	E-DEN	765.47	275.20	−61.13	− 103.77, −18.49	0.005	−5.17	−40.41, 30.07	0.773
Magnesium mg/d	DEN	251.99	75.09	Ref	Ref	Ref	Ref	Ref	Ref
	D-DEN	241.73	75.90	−10.26	−20.95, 0.43	0.060	−0.59	−7.99, 6.81	0.876
	E-DEN	207.96	68.91	−44.03	−54.87, −33.20	< 0.001	−17.72	−25.73, −9.71	< 0.001
Potassium mg/d	DEN	2725.90	789.24	Ref	Ref	Ref	Ref	Ref	Ref
	D-DEN	2772.58	799.12	−99.94	− 211.69, 11.80	0.080	0.19	−77.98, 78.35	0.996
	E-DEN	2448.16	753.88	− 424.36	− 537.58, − 311.14	< 0.001	− 151.27	− 235.85, −66.70	< 0.001
Iodine μg/d	DEN	187.44	95.70	Ref	Ref	Ref	Ref	Ref	Ref
	D-DEN	180.88	78.63	−6.56	−19.07, 5.95	0.304	−1.40	− 12.75, 9.95	0.808
	E-DEN	167.95	77.66	−19.50	−32.17, − 6.82	0.003	−5.22	− 17.50, 7.06	0.405

Data analysed using multiple regression. Data presented as means (SD), and unadjusted and adjusted differences in means (95% CI). ^a^Variables were naturally logged transformed for analysis, and presented as geometric means (IQRs), and ratio of the geometric means (95% CIs). DEN group was fixed as the reference category in each model. ^b^Adjusted for age, gender, SES (low, med & high), living status (living alone or not living alone), energy intake (kJ/d), survey year and smoking status (current smoker, past regular smoker & never smoked).

Table 5 depicts the odds for achieving dietary recommendations according to dental status. After adjusting for socio-demographic variables, energy intake (kJ/d), survey year and smoking status, the E-DEN group was less likely than the DEN group to achieve the 5-a-day recommendation for fruit and vegetables (*P* = 0.001), the 18 g/d of NSP (*P* = 0.039) and the 140 g/wk. of oily fish recommendation (P < 0.001). No differences were observed between the DEN and D-DEN groups.

**Table 5:** The likelihood of achieving dietary recommendations according to dental status

		Meeting recommendation		Unadjusted			Adjusted^b^		
Dietary recommendation	Dental status	n	%	OR	95% CI	P	OR	95% CI	P
Fruit and vegetables (5 portions daily)^a^	DEN	189	41.4	Ref	Ref	Ref	Ref	Ref	Ref
	D-DEN	99	32.5	0.68	0.50, 0.92	0.012	0.78	0.56, 1.07	0.123
	E-DEN	56	19.2	0.34	0.24, 0.47	< 0.001	0.51	0.35, 0.75	0.001
Red and processed meat (≤70 g daily)	DEN	283	62.1	Ref	Ref	Ref	Ref	Ref	Ref
	D-DEN	180	59.0	0.88	0.65, 1.18	0.399	0.81	0.59, 1.11	0.194
	E-DEN	171	58.6	0.86	0.64, 1.17	0.339	0.79	0.56, 1.11	0.168
Oily fish (140 g per week)	DEN	148	32.5	Ref	Ref	Ref	Ref	Ref	Ref
	D-DEN	75	24.6	0.68	0.49, 0.94	0.020	0.73	0.52, 1.03	0.072
	E-DEN	41	14.0	0.34	0.23, 0.50	< 0.001	0.44	0.29, 0.67	< 0.001
NSP (18 g daily)	DEN	88	19.3	Ref	Ref	Ref	Ref	Ref	Ref
	D-DEN	49	16.1	0.80	0.55, 1.18	0.256	0.97	0.63, 1.50	0.904
	E-DEN	23	7.9	0.36	0.22, 0.58	< 0.001	0.55	0.31, 0.97	0.039

NSP, non-starch polysaccharides. ^a^One portion is 80g. Data analysed using logistic regression. Data presented as unadjusted and adjusted OR (95% CI). DEN group was fixed as the reference category in each model. ^b^Adjusted for age, gender, SES (low, med & high), living status (living alone or not living alone), energy intake (kJ/d), survey year, smoking status (current smoker, past regular smoker & never smoked)

Table 6 shows disparities in biochemical measures of nutritional status according to dental status. In the unadjusted model, the E-DEN group compared with the DEN group had significantly lower levels of vitamin B6 PLP (*P* < 0.001), vitamin C (P < 0.001) and β-carotene (P < 0.001). These group differences in biochemical measures of nutritional status remained significant after controlling for socio-demographic variables, energy intake, survey year and health behaviours including nutritional supplement use and smoking status. There were no differences in biochemical measures of nutritional status between the D-DEN and DEN groups.

**Table 6:** The relationship between dental status and biochemical measures of nutritional status

				Unadjusted			Adjusted^b^		
Nutrient	Dental status	Mean	SD	Mean difference	95% CI	P	Mean difference	95% CI	P
Retinol μmol/L	DEN	1.92	0.49	Ref	Ref	Ref	Ref	Ref	Ref
	D-DEN	1.91	0.54	−0.01	−0.12, 0.10	0.863	−0.00	−0.11, 0.10	0.941
	E-DEN	1.85	0.55	−0.07	−0.18, 0.05	0.272	− 0.07	−0.19, 0.06	0.305
Vitamin B6 PLP n/mol/L^a^	DEN	41.20	26.55, 59.53	Ref	Ref	Ref	Ref	Ref	Ref
	D-DEN	40.12	25.00, 59.00	−1.03	−1.17, 1.11	0.688	1.01	−1.13, 1.14	0.907
	E-DEN	29.07	18.15, 43.45	−1.42	−1.63, − 1.23	< 0.001	−1.27	− 1.47, − 1.10	0.001
Vitamin B12 pmol/L	DEN	269.61	104.67	Ref	Ref	Ref	Ref	Ref	Ref
	D-DEN	269.86	99.64	0.25	−21.10, 21.59	0.982	2.74	−18.23, 23.72	0.797
	E-DEN	254.93	107.56	−14.68	−37.49, 8.13	0.207	−5.32	−29.19, 18.55	0.662
Vitamin C μmol/L	DEN	49.76	21.52	Ref	Ref	Ref	Ref	Ref	Ref
	D-DEN	51.70	21.40	1.94	−2.58, 6.45	0.400	2.91	−1.32, 7.13	0.177
	E-DEN	39.05	21.06	−10.71	−15.57, − 5.85	< 0.001	−6.42	− 11.26, − 1.58	0.009
25-OH-D nmol/L	DEN	44.34	22.10	Ref	Ref	Ref	Ref	Ref	Ref
	D-DEN	43.62	20.74	−0.72	−5.25, 3.80	0.754	0.25	−4.05, 4.56	0.909
	E-DEN	39.97	23.38	−4.37	−9.28, 0.53	0.081	−2.25	−7.22, 2.73	0.375
β-Carotene μmol/L^a^	DEN	0.43	0.28, 0.69	Ref	Ref	Ref	Ref	Ref	Ref
	D-DEN	0.41	0.27, 0.62	−1.05	−1.12, 1.02	0.145	− 1.04	−1.11, 1.03	0.293
	E-DEN	0.34	0.24, 0.52	−1.14	−1.23, − 1.06	< 0.001	−1.10	− 1.19, − 1.02	0.015
Ferritin μg/L^a^	DEN	80.41	43.00, 164.50	Ref	Ref	Ref	Ref	Ref	Ref
	D-DEN	82.07	44.25, 153.75	1.02	1.19, 1.24	0.836	1.08	−1.13, 1.30	0.448
	E-DEN	74.47	43.50, 143.50	−1.08	−1.19, 1.24	0.470	1.02	−1.22, 1.27	0.863

Data analysed using multiple regression. Data presented as means (SD), and unadjusted and adjusted differences in means (95% CI). ^a^Variables were naturally logged transformed for analysis, and presented as geometric means (IQRs), and ratio of the geometric means (95% CIs). DEN group was fixed as the reference category in each model. ^b^Adjusted for age, gender, SES (low, med & high), living status (living alone or not living alone), energy intake (kJ/d), survey year, smoking status (current smoker, past regular smoker & never smoked) and nutritional supplement use (yes/no).

## Discussion

The findings of this study showed that edentate, denture-wearing older adults living in the UK had lower daily intakes of important nutrients, and were less likely to achieve key dietary recommendations compared with dentate older adults. These findings were corroborated by the biochemical measures of nutritional status, as vitamin B6 PLP, vitamin C and β-carotene plasma concentrations were also lower in the edentate, denture-wearing group. On the contrary, there were no differences in biochemical measures of nutritional status, or nutrient intakes except for mean daily intake of calcium and carbohydrate, between the dentate, denture-wearing group and the dentate group (natural teeth, no dentures), after adjusting for socio-demographic and health behaviour variables. These results suggest that having no remaining natural teeth while wearing dentures increases the risk of reduced nutrient intake; while having natural teeth to support a prosthesis may result in nutrient intakes corresponding to those with only natural teeth and no prosthesis.

Despite the dentate, denture-wearing group having similar nutrient intakes to the dentate group, they did report, however, having difficulty eating more than half of the 11 selected foods. Individuals with removable partial dentures may find that their biting and chewing abilities are still less efficient than those with natural teeth. Suboptimal nutritional status, however, may not necessarily be a result of food avoidance due to difficulty eating, as particular foods in the diet can be replaced or modified to make them easier to consume, i.e. cooking carrots to soften their texture [22]. Without further investigations, such as undertaking a dietary pattern analysis, it is difficult to establish a reason for these conflicting findings.

The current study findings support previous cross-sectional studies that have also identified associations between impaired masticatory ability, food avoidance and reduced nutrient intakes [8–10]. Many previous large cross-sectional studies, however, have used retrospective methods, e.g. FFQ or a 24-h dietary recall, to assess dietary intake [9]. Although these are validated methods, they rely on memory, which may be an issue for some older adults, especially those with impaired cognition. A recent review of dietary assessment methods for examining the effect of nutrition on cognition, recommended using dietary records (food diary), which were used in the current study, to assess dietary intake of individuals with suspected memory problems, as they are completed at the time of eating [23].

The current study’s findings are also in accordance with prior research that also explored the relationship between dentition status and dietary intake using NDNS data collected between 1994 and 1995 [13, 14]. Over the past two decades, it appears that dental status has remained an important determinant of dietary intake in UK older adults, independent of socio-demographic and behavioural factors, and despite overall improvements in oral health status [7]. Although the proportion of edentate adults is reducing in the UK, edentulism is still prevalent among the oldest age groups [7] and those residing in care homes [24]. The current study highlights the need for strategies to help improve the nutritional status of edentate older adults, especially as diet plays an important role in protecting against age-related diseases.

Evidence from a recent systematic review indicated that prosthetic rehabilitation intervention studies replacing missing teeth do not necessarily prompt a dietary behaviour change [9]. Older adults’ dietary habits have likely evolved over a long time period, which presents a huge challenge when it comes to dietary behaviour change in this population. A combination of dental interventions focused on oral rehabilitation and dietary counselling/advice is the likely requirement for improving dietary intake in edentate older adults. To date only a small number of studies, especially RCTs evaluating these interventions have been undertaken. The limited evidence available, however, supports this type of intervention for improving fruit and vegetable intake in edentate adults [25, 26]. Future research is warranted in this area, especially as it is still unclear how these interventions should be designed, i.e. the theory used to guide the design of the intervention, the content of the intervention, the delivery (who delivers, where, how often etc.) and the behavioural change techniques that should be included.

The strengths of this study include the comprehensive approach undertaken to assess nutritional status that included measurements of dietary intake, body composition and biochemical indices of nutrient intakes [22]. Also, the range of data collected ensures the sample is well characterised with opportunity to adjust for a range of confounding factors in the analysis.

Several limitations, however, should be considered. Although NDNS is a large representative sample of the UK population living in private households, the survey excludes older adults residing in residential and long-term care facilities from sampling. The proportion of older adults who are edentate and have reduced functional dentition, however, are higher in care homes than in private households [24]. Furthermore, prevalence of malnutrition is high in care homes, with many residents having a BMI < 20 kg/m^2^ [27]. Future national dietary/dental surveys should include people residing in residential and long-term care facilities to ensure their sample is nationally representative.

Another limitation was that malnutrition was not adequately assessed in the NDNS RP, yet it has been estimated that one in ten people over the age of 65 years living in the UK are malnourished or at risk of malnutrition, and the majority live in the community [28]. Nevertheless, BMI was assessed in the NDNS RP, and recommended cut offs (< 20 kg/m^2^ if < 70 years and < 22 kg/m^2^ if > 70 years) were applied in the current analysis for the recognition of malnutrition [21]. The NDNS RP, however, should consider the addition of a malnutrition screening tool such as the Mini Nutritional Assessment (MNA) [29] or the Malnutrition Universal Screening Tool (MUST) [30] for future surveys, especially as the proportion of older adults in the UK is increasing.

Dietary intake information was collected using a self-reported method, which can be subject to social desirability bias, resulting in under- or over-reporting of energy intake, and subsequently nutrient intake. Although individuals with implausible energy intakes were excluded from the current analysis to account for misreporting, a crude method was used, which consequently may not have captured all implausible intakes. While biochemical measures of nutritional status were assessed, and supported the findings obtained from the self-reported dietary intake data; less than half the sample (515 out of 1053) provided a blood sample.

A weakness is that a large number of tests were conducted and therefore significant results should be interpreted with caution. Furthermore, this was a cross-sectional study; thus, a cause and effect relationship cannot be truly established. To date there has been a paucity of longitudinal studies undertaken that have examined the relationship between dentition status and nutrient intakes in older adults, which makes it difficult to determine the temporal order of events [31]. For instance, the relationship between dental status and nutrient intakes may be bidirectional, as consuming a poor quality diet can be a risk for tooth loss [32].

An oral health physical examination was not undertaken; hence, the oral health information collected by NDNS was self-reported and limited. Unfortunately, no information was collected regarding the number of natural teeth remaining, the design of the dentures used, or how well they fitted. However, physical examinations, including clinical oral assessments are expensive and time consuming to undertake in large cohorts. Oral health status appears to be an important determinant of nutrient intake in older adults; hence future national diet and nutrition surveys should consider utilising a clinical oral examination to capture detailed oral health information. Likewise, dental surveys should consider capturing detailed dietary intake information.

The findings from this study indicate that edentate older adults are still at a nutritional disadvantage compared to those with remaining natural teeth. Future research should focus on developing dental interventions coupled with dietary advice or counselling to encourage the adoption of healthy eating habits in this high-risk population group.

## Abbreviations

25-OH-D: 25 hydroxyvitamin D
BMI: Body mass index
CAPI: Computer assisted personal interview
CI: Confidence interval
D-DEN: Dentate with dentures
DEN: Dentate with no dentures
E-DEN: Edentate with dentures
NDNS RP: National Diet and Nutrition Survey Rolling Programme
NSP: Non-starch polysaccharides
NSSEC: National statistics socioeconomic classification
OR: Odd ratio
PLP: Pyridoxal 5 phosphate
PSU: Primary sampling unit
SES: Socioeconomic status
